# A novel 3D-printed patient-specific instrument based on “*H*-point” for medial opening wedge high tibial osteotomy: a cadaver study

**DOI:** 10.1186/s13018-022-03057-w

**Published:** 2022-03-18

**Authors:** Guo-Bin Liu, Sen Liu, Chao-Hua Zhu, Jia Li, Jun Li, Guo-Xing Jia, Wei Dong, Feng Zhao, Ye Huang

**Affiliations:** 1 Department of Orthopedics, The First Hospital of Hebei Medical University, Shijiazhuang, Hebei China; 2 Department of Joint Reconstructive Surgery, Beijing Jishuitan Hospital, NO. 31 Xinjiekou East Street, Beijing, 100035 China

**Keywords:** 3D printing, Patient-specific instrument, *H*-point, OWHTO, Cadaver study

## Abstract

**Background:**

Opening wedge high tibial osteotomy (OWHTO) is an effective surgical treatment for knee osteoarthritis. This study aimed to explore the feasibility and accuracy of a novel 3D-printed patient-specific instrument (PSI) based on “*H*-point” for medial OWHTO in a prospective cadaver study.

**Methods:**

Twenty-six fresh-frozen lower limbs were collected and randomly divided into two groups: PSI group treated with 3D virtual preoperative planning and a novel 3D-printed PSI; control group with the standard technique. 3D models were reversely reconstructed for preoperative surgical planning, guide plate design, and simulated osteotomy. Anatomic features of “*H*-point,” surgical time, fluoroscopic dose, correction accuracy including tibiofemoral angle (FTA) and posterior tibial slope (TS) angle were measured.

**Results:**

First, *H*-point was always described as a bony bulge in the posteromedial to the proximal tibia and had a relatively constant relationship with the osteotomy site. Second, the absolute correction error of mFTA and TS were significantly smaller in the PSI group. The effective rate of TS in the PSI group was more concentrated with absolute correction error within 1° and within 2° for 53.3% and 93.3%, compared to 9.1% and 45.5% in the control group. The total operation time, positioning osteotomy time, distraction correction time and fluoroscopy dose in the PSI group were significantly less than those in the control group.

**Conclusions:**

The novel 3D-printed PSI based on *H*-point is feasibility and accuracy with advantages in terms of TS, surgery time and radiation dose for OWHTO.

## Introduction

Since the introduction of the high tibial osteotomy (HTO) by Jackson and Waugh in the 1960s [1], knee-preserving surgery has recently been an increasingly popular surgical procedure to treat medial knee osteoarthritis (OA). HTO is typically indicated for an early or mild stage of knee OA, and the long-term efficacy has been published to be good or excellent in the overall treatments [2, 3]. Moreover, about 90% of young active patients can return to work or exercise within one year [4]. Medial opening wedge HTO is an increasingly popular procedure performed in most orthopedic departments worldwide with two major advantages for correcting the varus malalignment of the lower extremities and promoting the regeneration of articular cartilage potentiality [5]. The biomechanical basic principle is shifting the weight-bearing axis toward lateral, redistributing the stress on the whole knee, and reducing the excessive or discomfort burden of the medial compartment [6]. Nevertheless, for correction of varus malalignment of the lower extremities, conversion to actual operation appears to be a high technique-demanding in dealing with manual creation and distraction of HTO wedge [7]. Even though addressing the malalignment adequately, surgical procedures especially for inexperienced surgeons may lead to potential complications, such as fractures of the lateral cortex or tibial plateau, displacement of the lateral hinge, increased tibial slope, infections, functional disability and delayed union [8, 9].

Proper preoperative planning and precise surgical reproducibility have been considered to be the key factors by which opening HTO can obtain accurate correction results [10, 11]. To achieve correction accuracy, fluoroscopy methods are commonly used to measure HTO parameters based on the mechanical femorotibial Angle (mFTA) or weight-bearing line (WBL) [12, 13]. However, due to two-dimensional (2D) images, only sagittal plane data can be available preoperative, making it fairly challenging to determine the proper osteotomy opening distance without inadvertently altering other parameters such as the tibial slope or the extensor apparatus [14, 15]. In theory, minor inaccuracy of sawing direction or opening height in the coronal plane can lead to the 3D variation of the lateral hinge and proximal tibia even under the same correction angle in the sagittal plane, which hinders the long-term outcome and satisfaction of this operation [11, 16]. Excessive intraoperative radiation exposure remains another downside for this procedure. To achieve accurately angular correction in both the sagittal and coronal planes, the doctor and engineer have been dedicated to develop new equipment and new technology for modern OWHTO.

With the development and gradual sophistication of 3D printing technology, there emerged in succession various versions of this novel technology in the orthopedic field [17, 18]. The introduction and application of 3D-printed patient-specific instrumentation (PSI) in HTO surgery will be an important attempt for the treatment of knee osteoarthritis and may be a solution to the accuracy requirements of HTO planning and execution [19]. In the development of document retrieval for the digital library, the design of the existing PSI guide plate has been almost developed only based on the matching surface between the guide plate and the bone cortex. Recently, we have found a bony bulge in the posteromedial to the proximal tibia which was defined as “*H*-point” and observed to have a relatively constant positional relationship with the osteotomy site.

In this study, we will attempt to investigate and evaluate this new anatomical structure both in the 3D model and clinical operation. This study aims to introduce the basic concept, anatomic features and special clinical manifestations of the “*H*-point” and secondly explore the feasibility and accuracy of using this novel 3D-Printed PSI based on the “*H*-point” for medial OWHTO in a prospective cadaver-controlled study.

## Materials and methods

### Objects and groups

Twenty-six entire fresh-frozen lower limbs from thirteen cadavers were collected, and all specimens were provided by the anatomy laboratory of Hebei medical university. Ethics committee of the first hospital of hebei medical university approved this study (NO: 20210362). All specimens underwent roentgen graphic examination to exclude gross defects, congenital deformities, fractures and tumors. All specimens were performed with the CT-scan and radiography of the entire leg from the hip to the ankle. First, we attempted to search and present a new bone anatomic structure defined as “*H*-point” on the tibial developed for positioning reference of the PSI guide. And secondly, all lower limbs were randomly divided into two groups: PSI group (*n* = 15) treated with 3D virtual preoperative planning and a novel 3D-printed patient-specific instrument; control group (*n* = 11) with conventional X-ray planning and standard technique with freehand osteotomies. Position of “*H*-point”**,** anatomic features of “*H*-point,” surgical time, fluoroscopic time, correction accuracy including tibiofemoral angle (FTA) and posterior tibial slope (TS) angle were measured for statistics.

### Anatomic of “*H*-point”

First, the anatomic feature of “*H*-point” was evaluated in the proximal tibia 3D reconstruction model. The steps of 3D reconstruction were described in more detail in subsequent sections of “3D preoperative planning.” Based on experience, it is always the case in all models that there existed a bony bulge in the posteromedial to the proximal tibia. The vertical and horizontal distances from “*H*-point” to the medial edge of the tibial plateau, posterior edge of the tibial plateau, medial cutting point and *K*-wire fixation sleeve of No.3 were measured. Second, the skin rectangle incision centered at the condylus medialis femoris was performed for isolated medial exposure on adult cadaveric lower limbs. The skin and underlying tissues within the area of the incision were removed and muscle groups were exposed in turn. We needed to verify the existence of the *H*-point and further observe the relationship between *H*-point and the surrounding structure.

### Procedures for PSI guide applications

There are several generally basic procedures for PSI guide applications, including 3D preoperative planning, PSI guide plate design, simulated osteotomy, 3D printing and clinical application.

### 3D preoperative planning and simulated osteotomy

**Fig. 1:**
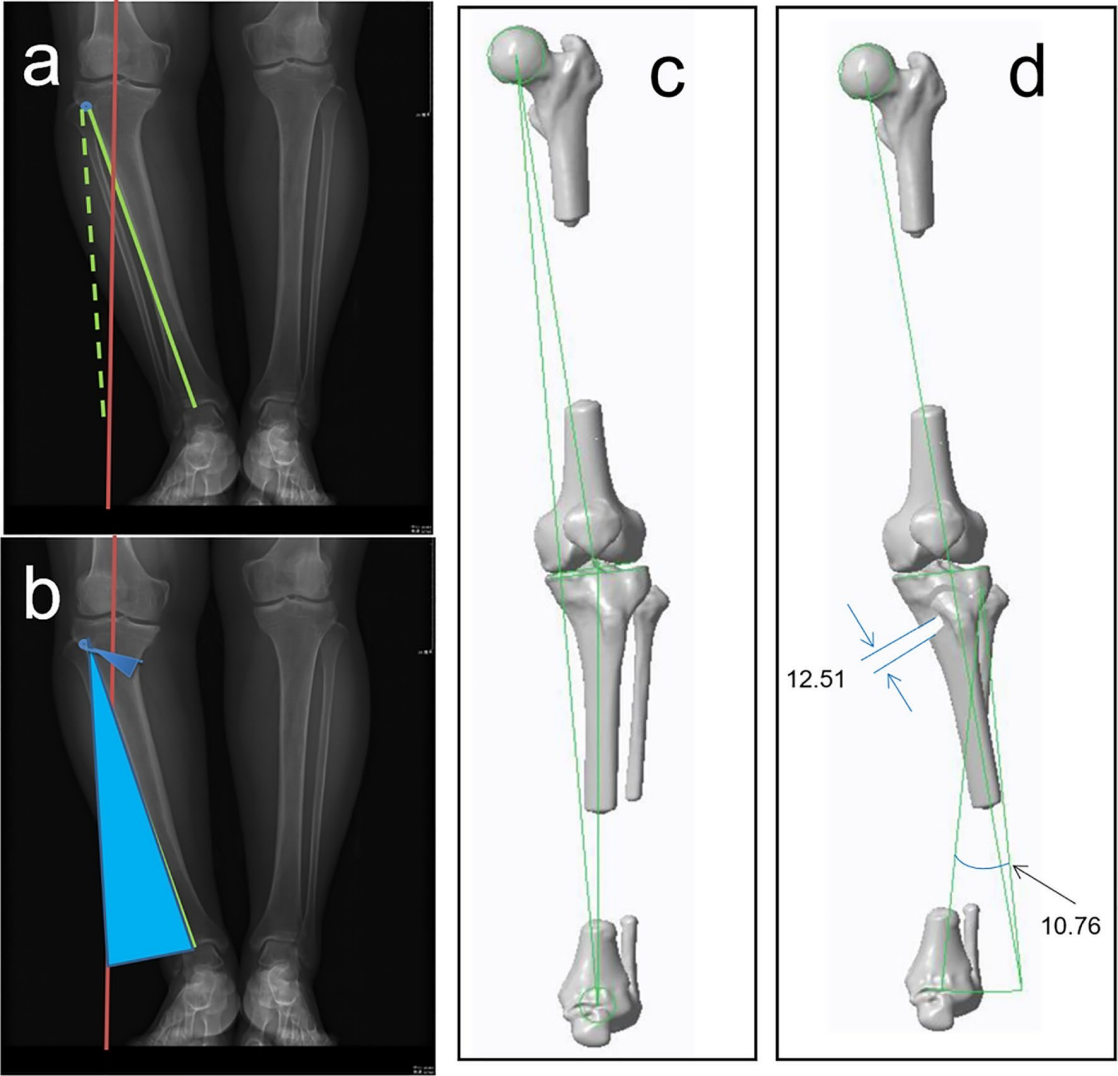
Simulated osteotomy. Lower limb 3D model was built with CT scanning data by medical image processing software and then loaded into the medical design software. The parameters of medial OWHTO were set including cutting point, lateral hinge, sawing direction, sawing depth, and correction angle. The distraction of the medial opening wedge was calculated by the "Miniaci” method according to the rotating degree of the target lower limb force line from the trigonometric perspective (**a**, **b**). The position of the lateral hinge was suggested to be about 5–10 mm from the lateral edge of the tibia plateau (**c**, **d**)

3D preoperative planning was accomplished by performing the following steps: (1) CT scanning of the full lower limbs ranging from the highest point of the hip to the lowest point of the ankle was executed with a slice separation of 5 mm and precision tomography of 0.5 mm around the knee joint within 15 cm range. (2) Lower limb 3D model was built with CT scanning data by medical image processing software (MIMICS 17.0) [20] and then loaded into the medical design software (SolidWorks 2018). The parameters of medial OWHTO were set including cutting point, lateral hinge, sawing direction, sawing depth, and correction angle. The target lower limb force line was uniformly and artificially designed target value of plan correction for increasing mFTA by 8° in valgus at baseline. The cutting point was set at 3–4 cm below the medial tibial plateau or the most concave part of the medial proximal tibia [21]. The position of the lateral hinge was suggested to be about 5–10 mm from the lateral edge of the tibia plateau [22]. The sawing direction is the line between the uppermost end of the fibular head and the medial cutting point and the sawing depth should be reserved for 1 cm width of the lateral hinge [23]. With the hinge as origin, the distal part of the osteotomy was valgus rotated in place around the lateral hinge relative to the proximal part until the lower limb force line transferred to the desired location. The distraction of the medial opening wedge was calculated by the “Miniaci” method according to the rotating degree of the target lower limb force line from the trigonometric perspective (Fig. 1). If the posterior tibial slope was changed in the simulated osteotomy, we should adjust the lateral hinge and height proportion of wedge distribution until it achieved the original slope.

### PSI guide plate design

**Fig. 2:**
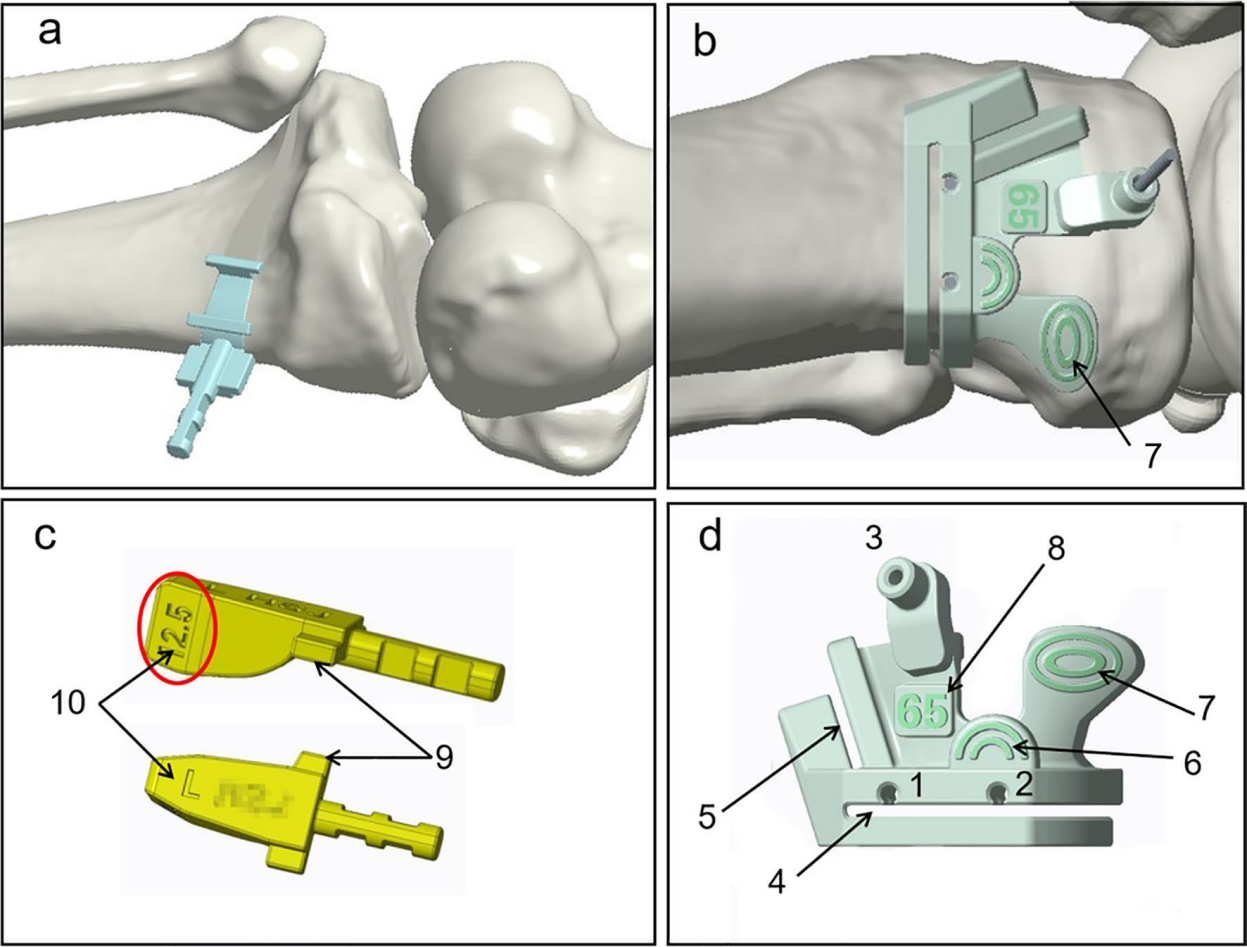
PSI guide plate design. Three Kirschner wire fixation sleeves (No.1, No.2, No.3) to stably fix the guide (**d-1**, **2, 3**); Two cutting slots including horizontal osteotomy groove (**d-4**) and ascending anterior osteotomy groove (**d-5**); Pressing point 1 **(b**, **d-7)**; Pressing point 2 (**d-6**); A wedge-shaped filling block designed independently (**c**); The shape of the wedge-shaped filling block was the same as target gap (**a**); A limited deep ear (**c-9**), the height of gap and direction marker (**c-10**) on the wedge-shaped filling block

The PSI guide was designed by Johnson & Johnson (Suzhou) Medical Co., LTD. Three *K*-wire fixation sleeves (No. 1, No. 2, No. 3) were designed to stably fix the guide. Among them, the position and direction of the *K*-wire fixation sleeve No.3 were the same as that of the *B* hole screw in the tomofix locking plate. Two cutting slots including horizontal osteotomy groove and ascending anterior osteotomy groove presented a 110-degree angle between them were provided for the biplanar osteotomy. Pressing point 1 defined “*H*-mark” matched with the *H*-point position. Pressing point 2 was used to assist guide plate stably fixation. Osteotomy depth was marked on the PSI guide to remind the surgeon. A wedge-shaped filling block was designed independently, the shape of which was the same as that of the target gap, and a limited deep ear was set to prevent the incorrect insertion. The end of the wedge-shaped filling block was designed according to the tomofix locking plate position, which could provide the correct fixed position of the plate together with the prefabricated *B* hole screw (Fig. 2).

### Cadaver application

**Fig. 3:**
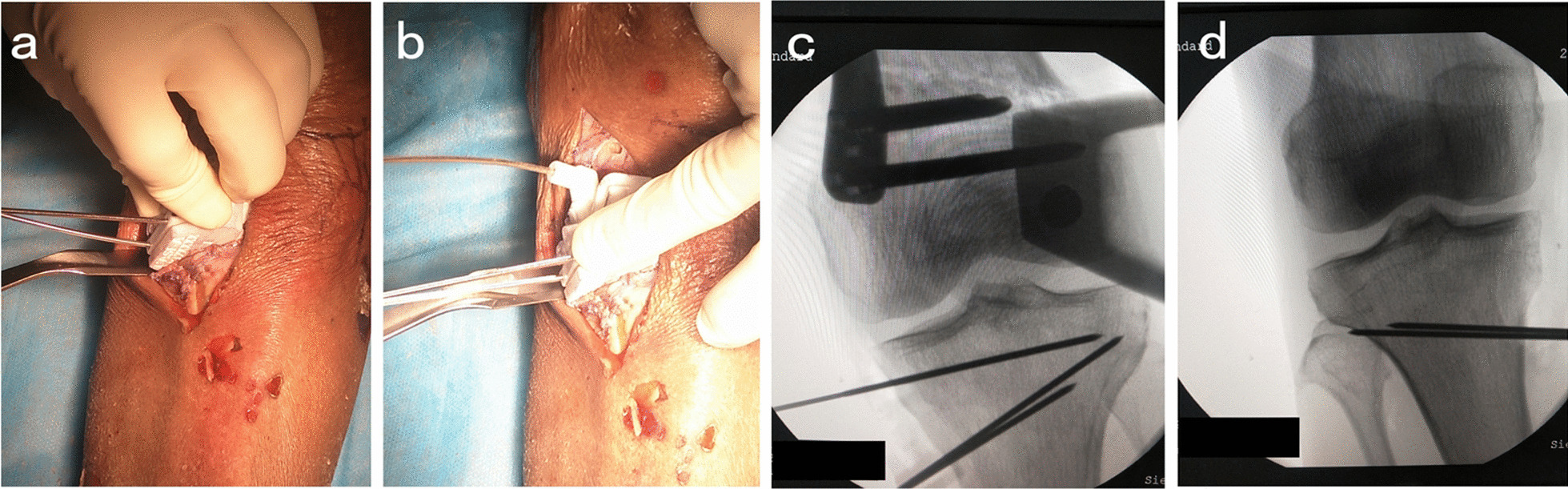
Insertion of *K*-wires. The *K*-wire was inserted through *K*-wire fixation sleeves (No. 1, No. 2, No. 3) (**a**, **b**). The *K*-wire (No.1, No.2) was inset at 3–4 cm below the medial tibial plateau. The position of the lateral hinge was suggested to be about 5–10 mm from the lateral edge of the tibia plateau. The *K*-wire (No.1, No.2) direction is the line between the uppermost end of the fibular head and the medial cutting point and the sawing depth should be reserved for 1 cm width of the lateral hinge (**c**, **d**)

**Fig. 4:**
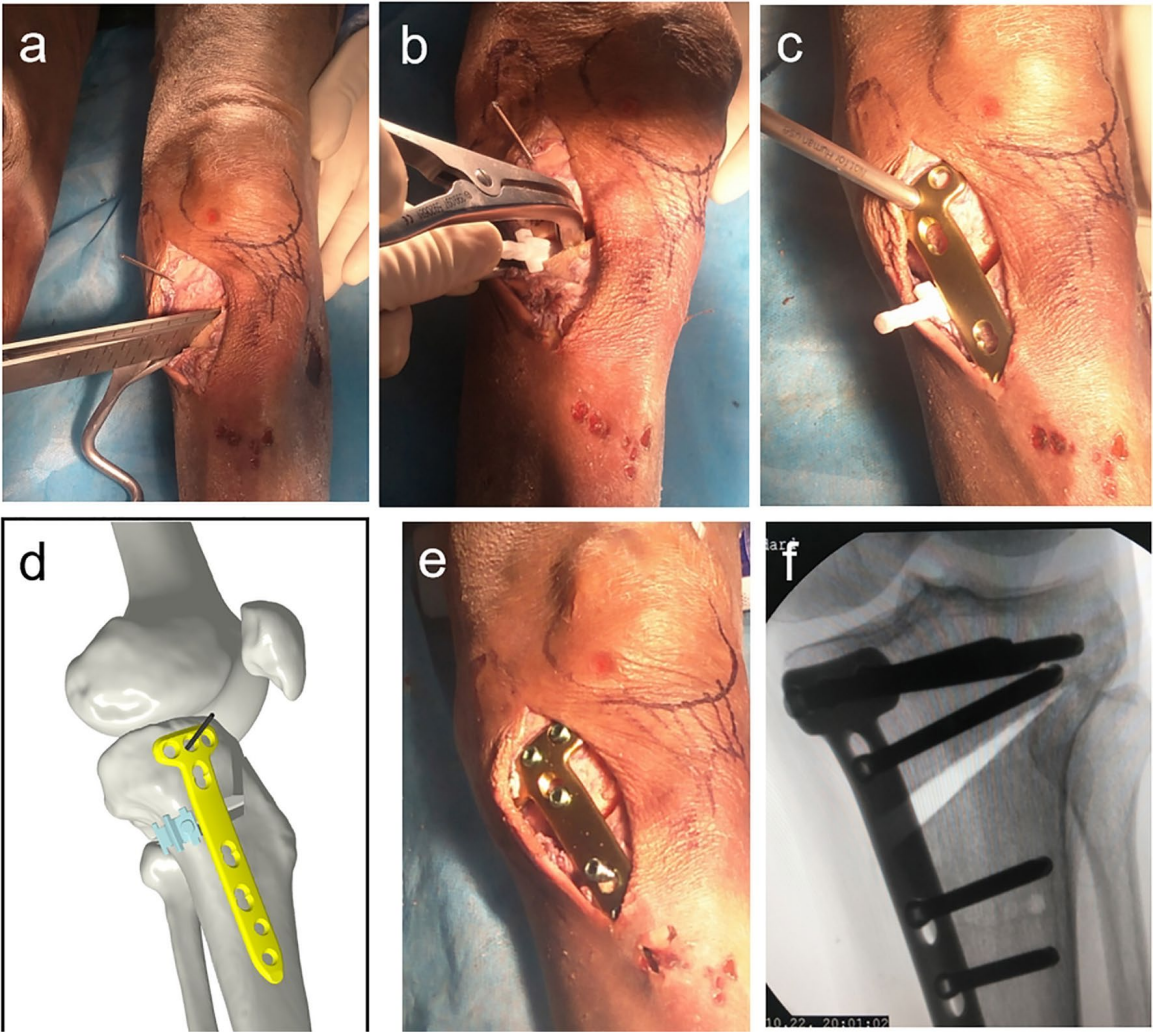
Distribution and fixation of wedge shape gap. PSI guide plate and guide needles of No. 1 and No. 2 were removed with guide needle of No. 3 retained a 3D printing wedge-shaped filling block was inserted to maintain the space (**a**, **b**); Guide needle of No. 3 retained and replaced by *B* hole screw in tomofix locking plate (**c**, **d**); The tomofix system was used to fix the locking steel plate, the position of which was determined by wedge-shaped filling block and *B* hole screw (**e**, **f**)

In the posterior 1/3 of the medial tibial plane, a longitudinal incision of about 8 cm was made from the level of the articular surface to the goose foot to peel off the medial soft tissue of the proximal tibia. The *H*-point was determined by hand at the medial posterior tibia about 2.5 cm below the joint line. The PSI guide plate was held by the medial tibial ridge from the rear. The pressing point 1 was close to the *H*-point and pushed toward the joint line. The pressing point 2 was close to the tibial bone surface and pressed vertically to ensure that the guide plate was completely attached to the bone surface. The *K*-wires were inserted through *K*-wire fixation sleeves to the posteromedial cortex in order of No. 1, No. 2 and No. 3. The positions of hinge and guide needles were confirmed by fluoroscopy to be consistent with the preoperative design. Cut short the *K*-wires, complete the ascending and horizontal osteotomy along the osteotomy groove of the guide plate, and control the depth of the saw blade according to the prompts of the guide plate. Then, the PSI guide plate and *K*-wires of No. 1 and No. 2 were removed with the *K*-wire of No. 3 retained. The wedge shape gap was widened length by length with steel rulers and fixed at the predetermined angle via a distractor. After this interspace was stretched, the steel rulers were removed and the 3D printing wedge-shaped filling block was inserted to maintain the space. The tomofix locking steel plate (Johnson & Johnson Medical Co., LTD) was used to fix, the position of which was determined by wedge-shaped filling block and *K*-wire of No.3. Finally, the *K*-wire of No.3 was replaced by a *B* hole screw (Figs. 3, 4).

### Statistical analysis

All statistical analyses were performed using SPSS software version 13.0 (SPSS, Inc., Chicago, IL). The results were expressed as the mean ± standard deviation (SD). One-way analysis of variance (ANOVA) was used for the analysis of Surgical time, fluoroscopic time, correction accuracy including tibiofemoral angle and posterior tibial slope angle, followed by Student–Newman–Keuls test for significant pairwise differences between subsamples. The statistical significant value was set at *P* < 0.05 in the univariate analyses.

## Results

### Basic characteristics of the specimen

Twenty-six entire fresh-frozen lower limbs from thirteen cadavers were collected and all lower limbs were randomly divided into two groups: PSI group (*n* = 15) with three-dimensional (3D) virtual planning and novel 3D-Printed Patient-Specific instrument; and control group (*n* = 11) with conventional X-ray planning and standard technique. All specimens were performed with the CT-scan and radiography of the entire leg from the hip to the ankle.

### “*H*-point” anatomic

**Fig. 5:**
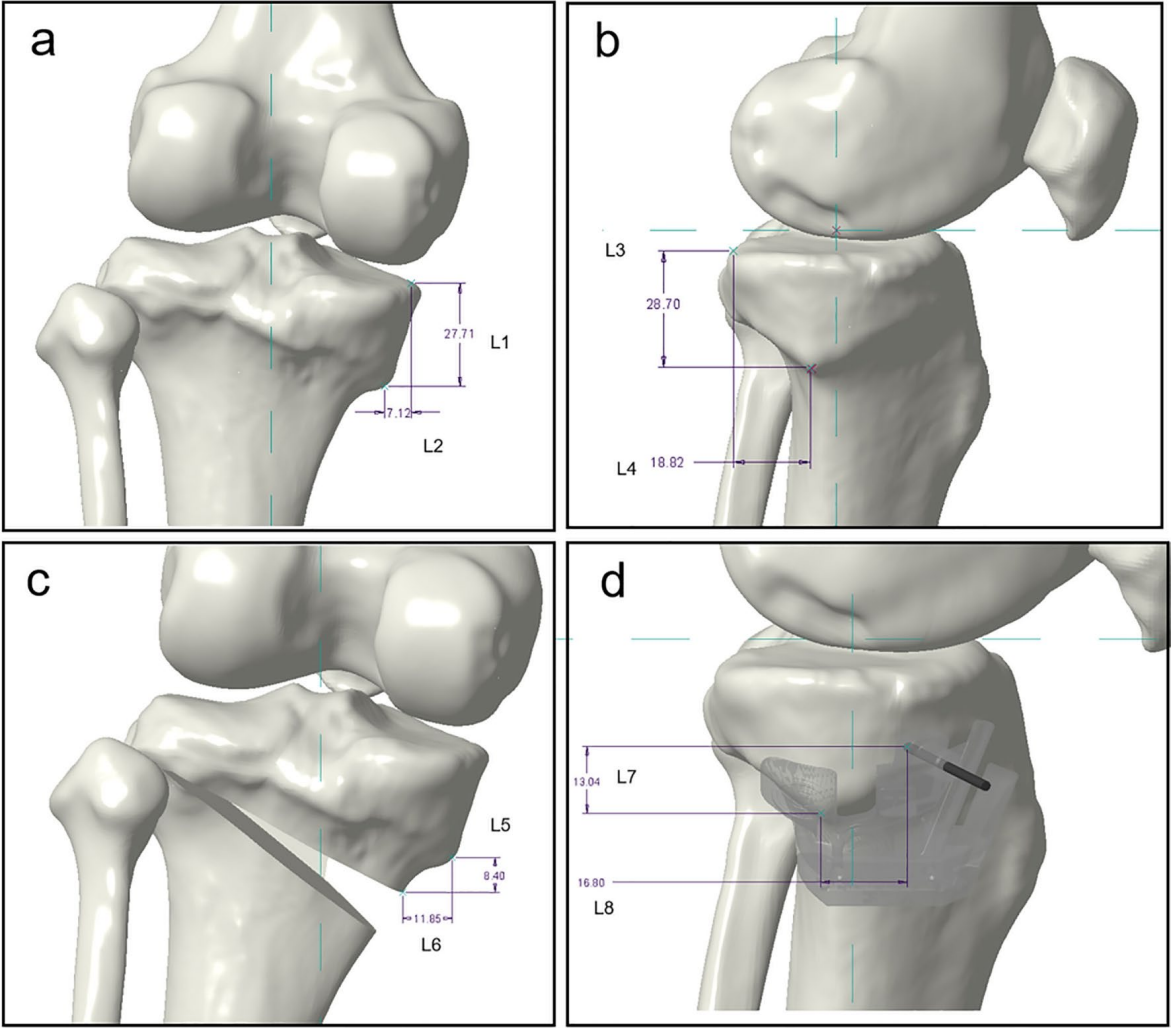
The measurement of *H*-point. The vertical and horizontal distances (mm) from “*H*-point” to the medial edge of the tibial plateau, posterior edge of the tibial plateau, medial cutting point and *K*-wire fixation sleeve of No. 3 were 7.12 and 27.71, 18.82 and 28.70, 11.85 and 8.40, 16.80 and 13.04

In 26 knees analyzed, the *H*-point was present. The measurement of the *H*-point on a 3D reconstruction model is shown in Table 1. The vertical and horizontal distances (mm) from “*H*-point” to the medial edge of the tibial plateau, posterior edge of the tibial plateau, medial cutting point and *K*-wire fixation sleeve of No. 3 were 7.16 ± 0.69 and 27.51 ± 1.6, 19.16 ± 1.20 and 27.60 ± 1.87, 11.62 ± 0.84 and 8.53 ± 0.59, 17.13 ± 1.68 and 12.86 ± 1.04, respectively. “*H*-point” could be used as a new anatomical marker for positioning the PSI guide (Fig. 5).

**Table 1:** The results of *H*-point measurement (*x* ± *s*)

*H*-point to	Vertical distance (mm)	Horizontal distance (mm)
Medial edge of the tibial plateau	7.16 ± 0.69	27.51 ± 1.64
Posterior edge of the tibial plateau	19.16 ± 1.20	27.60 ± 1.87
Cutting point	11.62 ± 0.84	8.53 ± 0.59
*K*-wire fixation sleeve of No.3	17.13 ± 1.68	12.86 ± 1.04

**Fig. 6:**
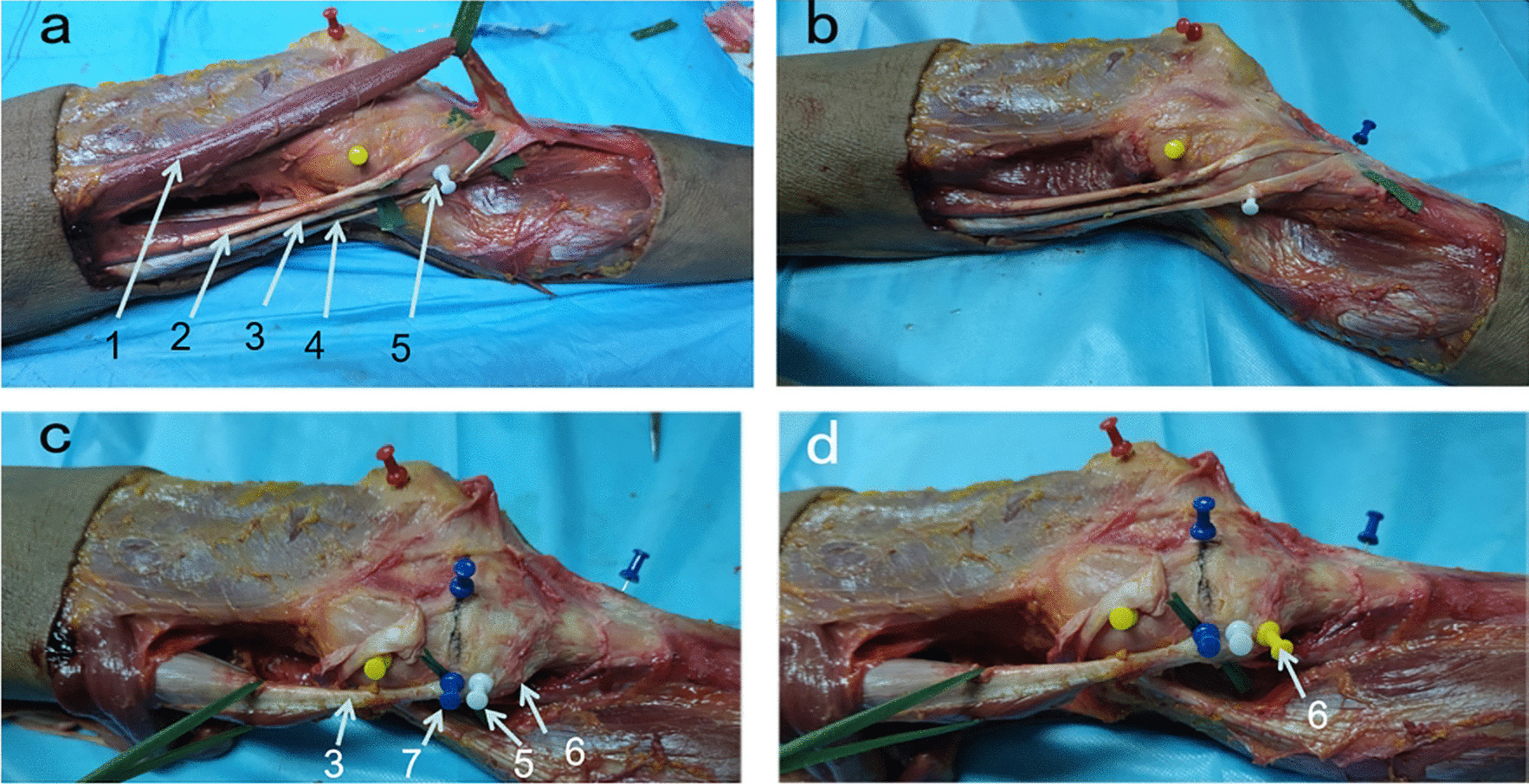
*H*-point anatomic. 1. Sartorius; 2. Graclils; 3. Semimembranous; 4. Semitendinosus; 5. Terminations of Semimembranous; 6. *H*-point; 7. The medial edge of the tibial plateau. The *H*-point was proved to be located above the terminations of the semimembranous muscle. The semitendinosus and gracilis tendons passed in proximity to the front of the *H*-point and ultimately formed the pes anserinus in the proximal tibia

We further verified the existence of *H*-point in specimen anatomical research as described in the 3D reconstruction model and observe the relationship between *H*-point and the surrounding structure. We found the bony bulge (*H*-point) which was proved to be located above the terminations of the semimembranous muscle. The semitendinosus and gracilis tendons passed in proximity to the front of the *H*-point and ultimately formed the pes anserinus in the proximal tibia (Fig. 6).

### Feasibility and accuracy of PSI guide applications

**Fig. 7:**
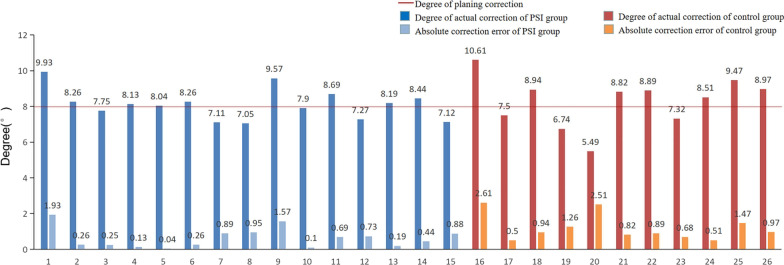
Comparison of mechanical femorotibial angle (FTA). Compared with the control group, the absolute correction error was significantly smaller in the PSI group indicating that the absolute correction accuracy of PSI was more concentrated

**Fig. 8:**
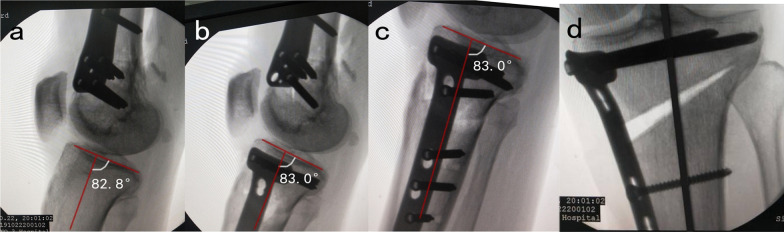
Comparison of posterior tibial slope (TS) angle. The PSI guide plate could effectively maintain the posterior tibial slope angle by the *B* hole screw and wedge-shaped filling block to accurately fix the locking plate in the planned position

- Before the operation, no significant difference was found in mFTA (178.13 ± 1.44 vs 179.23 ± 2.25, *P* = 0.143) and posterior tibial slope angle (TS) (8.92 ± 0.79 vs 8.72 ± 0.74, *P* = 0.520) between the two groups. Due to the cadaver study, the target lower limb force line was uniformly and artificially designed target value of plan correction for increasing mFTA by 8° in valgus based on the value already getting them. There was no significant difference in postoperative mFTA between the two groups (186.25 ± 1.47 vs 187.53 ± 2.33, *P* = 0.100) after the operation.
- The postoperative correction degree of mFTA was 8.11 ± 0.8 in the PSI group and 8.3 ± 1.42 in the control group, respectively, and the difference was not statistically significant (Fig. 7). However, compared with the control group, the absolute correction error was significantly smaller (0.62 ± 0.56 vs 1.20 ± 0.73, *P* = 0.032) in the PSI group indicating that the absolute correction accuracy of PSI was more concentrated for up to 86.7% within 1° error interval and 100% within 2°, compared with 63.6% and 81.8% in the control group, respectively **(**Table 2)
- For the comparison of posterior tibial slope angle (TS), a significant difference was found between the two groups after the operation (9.01 ± 1.43 vs 10.61 ± 1.35, *P* = 0.008) (Fig. 8). The absolute correction error of TS was smaller in the PSI group compared with the control group (1.24 ± 0.70 vs 2.18 ± 1.13, *P* = 0.015). And further analysis found that the absolute error in the PSI group was up to 53.3% within 1° error interval and 93.3% within 2°, compared with 9.1% and 45.5% in the control group, respectively, which indicated that the effective rate of TS maintenance in PSI group was more concentrated (Table 3).
- For comparison of operation time and fluoroscopy times, the total operation time, positioning time of osteotomy and distraction time of gap in the PSI group were significantly less than those in the control group; No significant difference was found in plate fixation time between the two groups. The total fluoroscopy times in the PSI group were controlled within 5 times on average, which was significantly less than that in the control group (Table 4).

**Table 2:** Comparison of mechanical femorotibial angle (x ± s)

	PSI group (*n* = 15)	Control group (*n* = 11)	*P*-value
Preoperative (°)	178.13 ± 1.44	179.23 ± 2.25	0.143
Postoperative (°)	186.25 ± 1.47	187.53 ± 2.33	0.100
Degree of planing correction (°)	8	8	
Degree of actual correction (°)	8.11 ± 0.84	8.3 ± 1.42	0.685
Absolute correction error (°)	0.62 ± 0.56	1.20 ± 0.73	0.032*
Absolute correction error within 1° (%)	86.7%	63.6%	
Absolute correction error within 2° (%)	100%	81.8%	

*Statistically significant difference (*P* < 0.05)

**Table 3:** Comparison of posterior tibial slope (TS) (*x* ± *s*)

	PSI group (n = 15)	Control group (n = 11)	*P*-value
Preoperative (°)	8.92 ± 0.79	8.72 ± 0.74	0.520
Postoperative (°)	9.01 ± 1.43	10.61 ± 1.35	0.008*
*P*-value	0.083	0.001	
Absolute correction error (°)	1.24 ± 0.70	2.18 ± 1.13	0.015*
Absolute correction error within 1° (%)	53.3%	9.1%	
Absolute correction error within 2° (%)	93.3%	45.5%	

*Statistically significant difference (*P* < 0.05)

**Table 4:** Comparison of surgical time and fluoroscopic times (*x* ± *s*)

	PSI group (*n* = 15)	Control group (*n* = 11)	*P*-value
Total time (min)	39.6 ± 4.35	54.59 ± 5.66	< 0.001*
Time of position and osteotomy (min)	15.4 ± 2.53	23.38 ± 3.89	< 0.001*
Time of gap distribution (min)	7.36 ± 1.12	16.73 ± 1.22	0.357
Time of fixation (min)	08.84 ± 1.06	9.09 ± 0.86	< 0.001*
Fluoroscopic times	4.07 ± 0.70	8.63 ± 2.06	< 0.001*

*Statistically significant difference (*P* < 0.05)

## Discussion

At present, limited by the shortcomings of 2D images [24, 25], traditional methods are still difficult for HTO to realize the accuracy and reproducibility of preoperative planning [26]. 3D-printed patient-specific instrumentation (PSI) initially implemented in maxillofacial surgery was proved to be an intraoperatively available customized surgical tool [27, 28]. So far, the clinical use of PSI in realignment procedures of HTO is still in a primary stage with only some macro-strategies and descriptive approaches and its true potential remains open-ended [26, 29].

In this study, our team had completed a PSI product concept design phase and then created a knee joint 3D model by reverse-engineering technology. In a 3D model, the anatomical structure of the surgical site could be visually and detailly displayed and selectively enlarged and rotated with higher accuracy and flexibility [30]. In the design process, HTO surgery was simulated in a 3D virtual environment. Theoretically, the 3D change of lateral hinge in the propping process of wedge osteotomy gap may influence tibial slope [11, 31]. With this unfavorable factor, the osteotomy plan must be adjusted to maintain the tibial slope if there is any change before and after the simulated operation [21]. Through 3D simulation osteotomy, the differences between preoperative planning and actual operation could be timely found and when varied greatly, doctors should assess the likelihood and impact of these consequences to avoid overcorrection [32]. According to HTO orthopedic principle, a few PSI for OWHTO have been reported in previous studies. Mao et al. [33] introduced a PSI guide plate designed two extended arms with two holes and an aligning rod to simplify the osteotomy gap opening process. However, this method required the design parameters from intraoperative and preoperative were completely consistent, which meant that there should be no mistakes in any step of the operation. If the hinge fracture or PSI guide plate was misplaced, the aligning rod would not work. Lukas et al. [34] designed a PSI guide with additional stabilizing hooks. Unfortunately, stabilizing hooks caused additional extensive periosteum dissection and the author did not evaluate the sagittal plane which was widely considered to be vulnerable to surgical failure [35, 36]. The findings showed that this novel PSI based on “*H*-point” could achieve more accurate osteotomy correction in both coronal and sagittal planes. PSI guide plate and wedge distraction module worked together to the coronal plane and guide needle of No. 3, distraction module and tomofix locking plate to the sagittal plane.

In addition, we have described a new anatomical marker-a bony bulge in the posteromedial to the proximal tibia defined as “*H*-point,” which was found to have a relatively constant positional relationship with the osteotomy site both in the 3D model and cadaver specimens. We have designed a unique 3D osteotomy guide plate. For the first time, we used the *H*-point as anatomical landmarks to guide the correct position of the PSI guide plate on the surface of the tibial cortex. Reference of *H*-point could provide doctors with more location information, make a more perfect match, and improve the surgeon's trust in the guide plate. Without the anatomic marker, a larger range of soft tissue dissection was needed for enough matching surfaces to avoid the position deviation of the guide plate, which might damage the medial collateral ligament and other structures. Our incision in our study could be reduced to an average of 8 cm compared to 10 cm reported by Mao [33]. Perez [10] and Kim [37] designed 3D printing wedge-shaped distraction module, trying to solve the soft tissue dissection by limiting PSI only to the osteotomy gap. Genechten et al. [20] reported that the accuracy of the coronal plane was effectively controlled when used 3D printing osteotomy module. However, the posterior tibial slope was significantly increased (2.1 ± 2.6 degrees). The posterior tibial slope would change during the coronal plane correction process of OWHTO [38, 39], which might lead to limited extension and tension overload of the anterior cruciate ligament. Our PSI guide plate could effectively maintain the posterior tibial slope angle by guide the needle of No. 3 and wedge-shaped filling block to accurately fix the locking plate in the planned position. The average level of fluoroscopy in our study was 5 times in the PSI group and 9 times in the control group during the whole osteotomy procedure, which was lower compared to routine clinical operation on a patient for almost 20–30 times. The difference among objects was probably the main reason for the experiment results above. The advantage of using cadaveric research was that we could attempt to pursue the smallest process to achieve the best results by verifying and simplifying some non-essential procedures according to satisfactory surgical results. Compared with cadaveric research, clinical surgery object was real patients, and due to small tolerance for errors, almost every key step was required to be confirmed by X-ray. So the more times of fluoroscopy must be considered as an unfavorable alternative. It must be emphasized that the appropriate fluoroscopy time for this novel PSI guide plate used in clinical practice needs to be further explored.

There were still some limitations in this study. The sample size was relatively small, so we needed to expand the sample size later. During the operation, we ignored the influence of the PSI guide plate on vascular and nerve tissue. We should pay attention to the further clinical trials for lacking long-term follow-up data of cadaver studies. Establishing a complete theoretical system of 3D printing PSI guide-assisted HTO would be a momentous event.

## Conclusion

The *H*-point had a relatively constant positional relationship with the osteotomy site. And the novel 3D-printed PSI based on the *H*-point is feasibility and accuracy with advantages in terms of posterior tibial slope, surgery time and radiation dose for OWHTO.

## Data Availability

The datasets generated and analyzed during the current study are availabled from the corresponding author on reasonable request.
